# β-Cyclodextrin Inclusion Complexes of *Cinnamomum camphora* Essential Oil: A Comparative Study on Encapsulation Strategies, Physicochemical Stability, and Cytotoxic Profile

**DOI:** 10.3390/pharmaceutics18010117

**Published:** 2026-01-16

**Authors:** José Adão Carvalho Nascimento Júnior, Anamaria Mendonça Santos, Ana Maria Santos Oliveira, Cláudio Carvalho Santana Júnior, Saravanan Shanmugam, Antonella Osses Toledo, Natalia Juica, Mikele Cândida Sousa de Sant’Anna, Adriano Antunes de Souza Araújo, Luis Constandil, Jeffri S. Retamal, Mairim Russo Serafini

**Affiliations:** 1 Postgraduate Program in Health Sciences, Federal University of Sergipe, Aracaju 49060-100, SE, Brazil; 2 Laboratory of Pharmaceutical Assays and Toxicity (LeFT), Federal University of Sergipe, São Cristóvão 49107-230, SE, Brazil; 3 Postgraduate Program in Pharmaceutical Sciences, Federal University of Sergipe, São Cristóvão 49107-230, SE, Brazil; 4 Department of Botany, Gobi Arts and Science College, Gobichettipalayam 638453, TN, India; 5 Laboratory of Neurobiology, Department of Biology, Faculty of Chemistry and Biology, University of Santiago of Chile, Santiago 9170124, Chile; 6 Center for Research in Bioeconomy, Environment, Innovation, Intelligence, Technology, Education and Health (BAITES), Federal University of Maranhão, São Luís 65085-580, MA, Brazil

**Keywords:** *Cinnamomum*, cyclodextrin, essential oil, inclusion complexes

## Abstract

**Background/Objectives**: Essential oils (EOs) from plants of the genus *Cinnamomum* have been widely used based on their antimicrobial, antioxidant, and anti-inflammatory properties. However, their elevated volatility and limited aqueous solubility restrict their use in pharmaceutical and food formulations. Cyclodextrins (CDs) have emerged as a promising strategy to overcome these limitations through the formation of inclusion complexes. **Methods**: In this study, inclusion complexes of essential oil from *C. camphora* L. (EOCNM) with β-cyclodextrin (β-CD) were developed using physical mixing (PM), ultrasonic treatment (US), and freeze-drying (FD). The inclusion complexes were physicochemically characterized by differential scanning calorimetry (DSC), thermogravimetric analysis (TG/DTG), X-ray diffraction (XRD), and scanning electron microscopy (SEM) to evaluate their physicochemical interactions and complexation efficiency. **Results**: Our results demonstrated successful complex formation, with the FD and US methods showing greater amorphization and stronger inclusion characteristics compared to the PM method. Thermal analysis confirmed improved physicochemical stability of the essential oil when complexed with β-CD. Furthermore, the cytotoxicity assay of the complexes was assessed using the MTT assay and J774 macrophage cells. The complexes exhibited low cytotoxicity, indicating their potential biocompatibility for biomedical and food applications. **Conclusions**: Overall, β-CD encapsulation effectively enhanced the physicochemical stability and safety profile of *C. camphora* essential oil, providing a promising strategy for its controlled delivery and protection against degradation.

## 1. Introduction

Essential oils (EOs) are complex mixtures of volatile, low-molecular-weight compounds synthesized by aromatic plants as secondary metabolites. They have been utilized in traditional medicine, cosmetics, and food preservation due to their broad range of biological activities, including antimicrobial, antioxidant, anti-inflammatory, and analgesic effects [1,2,3]. In particular, essential oils obtained from *Cinnamomum camphora* L. (camphor tree) produce an essential oil rich in monoterpenes such as camphor, as well as terpenes, phenols, flavonoids, alkaloids, and coumarins [4,5]. These compounds have been reported to possess significant antimicrobial, anti-inflammatory, and antioxidant properties, supporting the traditional use of *C. camphora* oil in topical and inhalation therapies [4,6]. Thus, *C. camphora* and its derivatives have attracted considerable interest for potential pharmaceutical, cosmetic, and food applications [7].

Despite their recognized therapeutic potential, essential oils exhibit several physicochemical limitations, including high volatility, poor water solubility, and instability when exposed to light, heat, or oxygen [8]. These limitations often result in rapid degradation, evaporation, and reduced bioavailability, compromising their efficacy and shelf life [9,10]. To overcome these limitations, encapsulation strategies have been developed to protect volatile compounds, improve their water solubility, and enhance their stability and bioavailability.

Cyclodextrins (CDs) have gained significant attention due to their ability to form inclusion complexes with hydrophobic molecules through noncovalent interactions within their hydrophobic cavities [11,12]. β-CD, composed of seven α-1,4-linked glucose units, is the most widely used cyclodextrin due to its cavity size, low cost, and biocompatibility [13]. Complexation with β-CD has improved solubility, protected active compounds from degradation, controlled release, and enhanced thermal and chemical stability, making it particularly attractive for pharmaceutical, cosmetic, and food applications [14,15,16].

Recent studies demonstrated the effectiveness of β-CD inclusion complexes in enhancing the stability and biological performance of essential oils and their constituents [17,18]. These findings highlight the use of CD complexation as an effective approach to optimize the physicochemical and pharmacological properties of essential oils [19,20]. Different methodologies have been proposed for the preparation of EO/β-CD inclusion complexes, including physical mixing (PM), co-precipitation, ultrasound-assisted complexation (US), and freeze-drying (FD). The efficiency of each technique depends on variables such as temperature, solvent system, and energy input, which influence molecular interactions and the structural organization of the complex [21]. Therefore, a comparative assessment of these preparation methods is essential to optimize encapsulation efficiency and biological performance.

In this study, we aimed to develop and characterize β-CD inclusion complexes containing *Cinnamomum camphora* essential oil (EOCNM) using PM, US, and FD complex techniques. The obtained complexes were analyzed through differential scanning calorimetry (DSC), thermogravimetric analysis (TG/DTG), X-ray diffraction (XRD), and scanning electron microscopy (SEM) to evaluate their structural and thermal properties. In addition, cytotoxicity was assessed using the MTT assay in murine macrophage (J774) cells to determine biocompatibility. This study examines the impact of various encapsulation methods on the physicochemical properties and biological performance of EOCNM/β-CD inclusion complexes, offering insights into their optimized application in pharmaceutical and food formulations.

## 2. Materials and Methods

### 2.1. Preparation of C. camphora Essential Oil/β-CD Inclusion Complexes

The samples were prepared using two distinct methods, ultrasound and freeze-drying, as previously described [22]. Briefly, the inclusion complexes were formulated at a 1:1 molar ratio based on the molecular weights of camphor (152.23 g·mol^−1^) and β-CD (1135.0 g·mol^−1^, purchased from Sigma-Aldrich, St. Louis, MO, USA, purity ≥ 97%). All formulation preparations were carried out at room temperature (≈25 °C, 1 atm) to minimize loss of EOCNM (purchased from Via Aroma, Porto Alegre, RS, Brazil, purity ≥ 99%). After preparation, samples were stored in glass desiccators at room temperature until physicochemical and cytotoxic characterization. For comparison, a PM of EOCNM and β-CD was prepared in a 1:1 molar ratio by adding the essential oil to β-CD powder in an agate mortar. The mixture was manually homogenized for 10 min until a homogeneous mixture was obtained and then stored in airtight amber glass containers within an electronic desiccator (Costar, model Desiccator DCV040, Aracaju, SE, Brazil) to prevent moisture absorption.

#### 2.1.1. Ultrasound (US) Formulation Procedure

For the US method, the essential oil (EOCNM, 152 mg) and β-CD (1135 mg) were each dissolved in 100 mL of ethanol and 100 mL of distilled water, respectively. The EOCNM solution was slowly added to the β-CD solution under continuous stirring at room temperature. The resulting mixture was subjected to ultrasonic bath (UltraCleaner 100 v, 40 kHz, 1000 watts, Solidsteel, Citta’ Sant’Angelo, Italy) treatment for 60 min, after ultrasonic treatment, the resulting aqueous EOCNM:β-CD dispersion was subjected to organic solvent removal under vacuum. The solution was then filtered through a 0.45 μm membrane, frozen at −4 °C, and lyophilized for 48 h at −50 °C under a pressure of 1.09 Pa using a lyophilizer, enabling the sublimation of the frozen aqueous phase and preservation of the inclusion complex structure (Labconco FreeZone 4.5, New York, NY, USA) [21].

#### 2.1.2. Freeze-Drying (FD)

For the FD method, 20 mL of distilled water was added to a container containing 150 mg of EOCNM and 1135 mg of β-CD. The mixture was stirred continuously at room temperature and equilibrated in an orbital shaker (Quimis Q 261A21, Quimis, Diadema, SP, Brazil) at 150 rpm for 36 h. After equilibration, the solution was frozen at −4 °C and lyophilized at −50 °C under a pressure of 1.09 Pa for 48 h using a lyophilizer (Labconco FreeZone 4.5, MSE Supplies, Tucson, AZ, USA).

### 2.2. Characterization of Inclusion Complexes

#### 2.2.1. Thermal Analysis

Thermal characterization of the inclusion complexes was performed using DSC and TG/DTG analyses, as previously described by Serafini et al. [15]. DSC analyses were conducted using a DSC-60A calorimeter (Shimadzu, Columbia, MD, USA). Approximately 2 mg of each sample was sealed in aluminum crucibles (Al) and analyzed under a dynamic nitrogen atmosphere (50 mL·min^−1^) at a heating rate of 10 °C·min^−1^ over a temperature range of 25–500 °C. The DSC cell was calibrated with indium and zinc standards prior to measurement.

TG/DTG analyses were performed using a TGA-60 thermobalance (Shimadzu, Columbia, MD, USA). Approximately 3 mg of each sample was placed in platinum crucibles (Pt) and heated from 25 °C to 900 °C under a dynamic nitrogen atmosphere (100 mL·min^−1^) at a heating rate of 10 °C·min^−1^. The TG/DTG system was calibrated using calcium oxalate monohydrate (CaC_2_O_4_·H_2_O) in accordance with ASTM standards [22].

#### 2.2.2. Scanning Electron Microscopy (SEM)

The surface morphology of pure β-CD and the inclusion complexes obtained by physical mixing, ultrasound, and freeze-drying methods was examined by the scanning electron microscope (JEOL JSM-7410F, JEOL, Akishima, Tokyo, Japan). The powders were previously fixed to a brass stub using double-sided adhesive tape and then made electrically conductive by coating them in a vacuum with a thin layer of gold for 60 s. The pictures were taken at an excitation voltage of 1 kV with copper filaments, at magnifications of 250× and 500× [23].

#### 2.2.3. X-Ray Diffractometry (XRD)

The X-ray powder diffraction patterns were obtained with a Rigaku diffractometer (Siemens D5000, Malvern, London, United Kingdom) equipped with a Ni-filtered Cu-Kα radiation source, a voltage of 40 kV, and a current of 40 mA. Data were collected over 2θ = 3.00–65.00° (θ being the angle of diffraction), with an accelerating voltage of 40 kV, in step-scan mode at 1 s^−1^. Samples were freeze-dried, and then 10 mg of each sample was added to the slide for packing prior to X-ray scanning [24].

### 2.3. Cytotoxicity Assay

The cytotoxicity of EOCNM was evaluated using the standard spectrophotometric 3-(4,5-dimethylthiazole-2-yl)-2,5-diphenyltetrazolium bromide (MTT, Thermo Fisher^®^, Waltham, MA, USA) assay [25]. MTT was resuspended in 500 μL of saline solution (0.9% NaCl), stirring for 1 min and ultrasonic bath (UltraCleaner 100 v, 40 kHz, 1000 watts, Solidsteel, Citta’ Sant’Angelo, Italy) for 15 min. The resulting suspensions were filtered through a 0.22 μm Millex filter (Merck Millipore, Sigma-Aldrich^®,^ St. Louis, MO, USA) and used to prepare sample dilutions.

J774G8 macrophage-like cells (ATCC, Monocyte-macrophage; Product code: ATCC-TIB-67) were cultured at 37 °C in a humidified atmosphere containing 5% CO_2_, using Dulbecco’s Modified Eagle’s Medium (DMEM, Sigma-Aldrich, St. Louis, MO, USA) supplemented with 10% fetal bovine serum (FBS, Sigma-Aldrich, St. Louis, MO, USA). To split the cells, after washing them with phosphate-buffered saline (PBS 1X), cells were trypsinized (Trypsin-EDTA 0.25%, Gibco, Frederick, MD, USA), centrifuged, and resuspended in 1 mL of medium. Viable cells were counted using a Neubauer chamber with trypan blue staining and then seeded into transparent, flat-bottom 96-well plates at 2.5 × 105 cells/well.

After a 24 h adhesion period, treatments with free EOCNM and the inclusion complex (EOCNM–β-CD) by the US method were applied at EOCNM-equivalent concentrations of 50 and 100 μg·mL^−1^. Triton X-100 (0.5% *v*/*v*) was used as a positive control for cytotoxicity, whereas untreated cells maintained in DMEM with 10% FBS served as the viability control. Following 72 h of incubation with the different compounds, MTT solution (0.5 mg·mL^−1^ in PBS) was added to each well 4 h before the end of the incubation period. Crystal formation was visually confirmed at the bottom of the wells; crystals were then solubilized in DMSO, yielding a purple solution indicative of viable cells, unlike wells treated with Triton X-100, where no MTT reduction occurred [26]. The product obtained was quantified spectrophotometrically by measuring absorbance at 570 nm using a SpectraMax^®^ M5 plate reader (Molecular Devices, San Jose, CA, USA), and cell viability was expressed as a percentage relative to the untreated group.

### 2.4. Statistical Analysis

Statistical analyses were performed using GraphPad Prism (GraphPad 10 Software, La Jolla, San Diego, CA, USA). Data were analyzed by two-way analysis of variance (ANOVA) followed by Tukey’s post hoc test. Results are expressed as mean ± standard deviation (SD). Statistical significance was indicated as follows: * *p* < 0.05, ** *p* < 0.01, and *** *p* < 0.001, where *p* > 0.05 was considered not significant (N.S.).

## 3. Results and Discussion

### 3.1. Complexation of C. camphora Essential Oil (EOCNM) in β-Cyclodextrin (β-CD)

**Figure 1:**
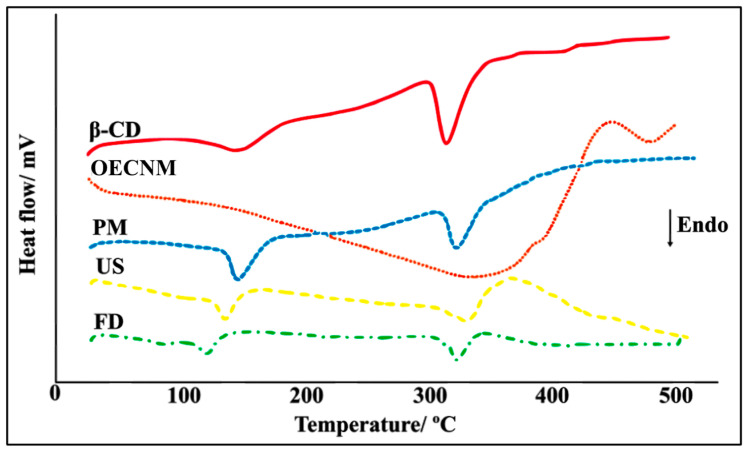
DSC curves in the dynamic N2 atmosphere of β-CD, EOCNM, PM, US, and FD.

The DSC curve of β-CD displayed two distinct endothermic events (Figure 1). The first event, observed within the temperature range of 109–177 °C, corresponds to the release of water molecules associated with β-CD (dehydration). The second event, occurring between 295 and 355 °C, corresponds to the melting of the β-CD molecule, followed by thermal decomposition and the elimination of the carbonaceous residue [27]. In contrast, the DSC thermogram of EOCNM exhibited a broad endothermic event between 100 and 390 °C, associated with volatilization and subsequent thermal decomposition of the oil components.

The DSC thermogram of the PM of β-CD and EOCNM displayed two endothermic events followed by decomposition (Figure 1). The first event, observed between 121 and 171 °C, is likely related to the evaporation of bound water in β-CD and to partial volatilization of oil molecules adsorbed on the surface. The second event, occurring between 295 and 340 °C, corresponds to β-CD degradation [28].

Conversely, the DSC curves of inclusion complexes prepared by US and FD methods showed significant differences in their thermal profiles compared with those of the pure components, indicating stronger molecular interactions and successful complexation. For the FD complex, two endothermic transitions were observed at 93–135 °C and 295–340 °C (Figure 1). In contrast, the DSC curve of the US complex lacked the volatilization peak typically associated with the essential oil, suggesting that the oil molecules were effectively encapsulated within the β-CD cavity.

### 3.2. Thermal Stability Analysis (TG/DTG)

**Figure 2:**
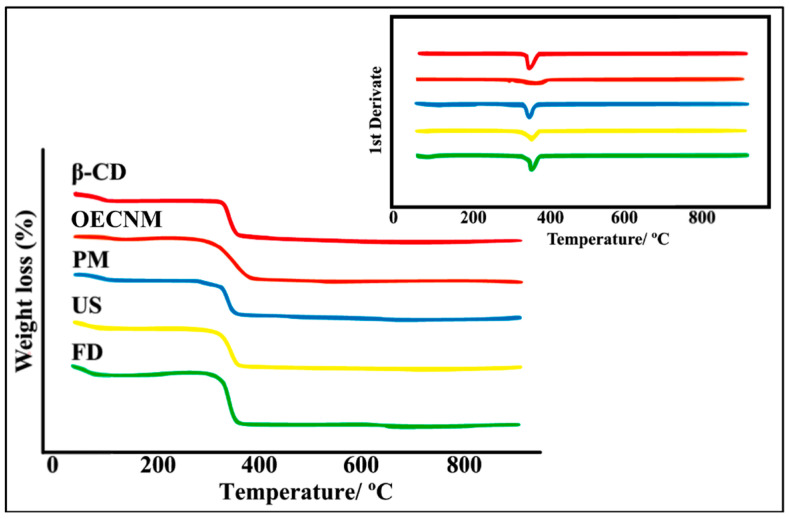
TG/DTG curves of β-CD, EOCNM, PM, US, and FD.

The thermal stability of EOCNM, β-CD, PM, and the inclusion complexes obtained by US and FD was evaluated using TG/DTG. The corresponding degradation profiles and temperature data are presented in Figure 2 and Table 1. Mass losses were analyzed across specific temperature intervals for each sample, revealing distinct degradation behaviors associated with the preparation methods. The TG/DTG curves of EOCNM exhibited a significant mass loss between 100 and 360 °C (Δm_2_ = 75.5 ± 0.32%), attributed to volatilization, followed by an additional loss of 14.4 ± 0.11% in the range of 360–600 °C (Δm_3_, Figure 2, Table 1), with a degradation temperature of approximately 260 °C.

For β-CD, four distinct stages of mass loss were identified. The first stage, occurring below 100 °C (Δm_1_ = 13.1 ± 0.22%), corresponds to dehydration, as water molecules are released from the β-CD cavity [28,29]. The second stage (100–360 °C), with a 74.6 ± 0.35% weight loss, represents the primary thermal degradation process, with decomposition occurring around 290 °C. The third stage (360–600 °C) resulted in a further 10% loss, and the fourth (600–900 °C) corresponded to the removal of residual carbonaceous material (1.5 ± 0.20%) (Figure 2, Table 1).

The TG/DTG profile of the PM largely overlapped with β-CD. The first mass loss (Δm_1_ = 13.4 ± 0.25%) occurred between 30 and 100 °C and was attributed to the release of water molecules from β-CD cavities (Figure 2, Table 1). In the subsequent stage, the PM displayed only minimal additional mass loss, confirming that this preparation method is ineffective for forming stable inclusion complexes between β-CD and the essential oil [28]. In contrast, both US and FD complexes demonstrated reduced mass loss in the initial stage (Δm_1_ = 11.1 ± 0.18% and 11.9 ± 0.24%, respectively) compared with PM, suggesting that essential oil components replaced some of the water molecules within the β-CD cavities (Figure 2, Table 1). The third (360–600 °C) and fourth (600–900 °C) stages correspond to the thermal decomposition and removal of the carbonaceous residues of β-CD [30].

### 3.3. Scanning Electron Microscopy (SEM)

**Figure 3:**
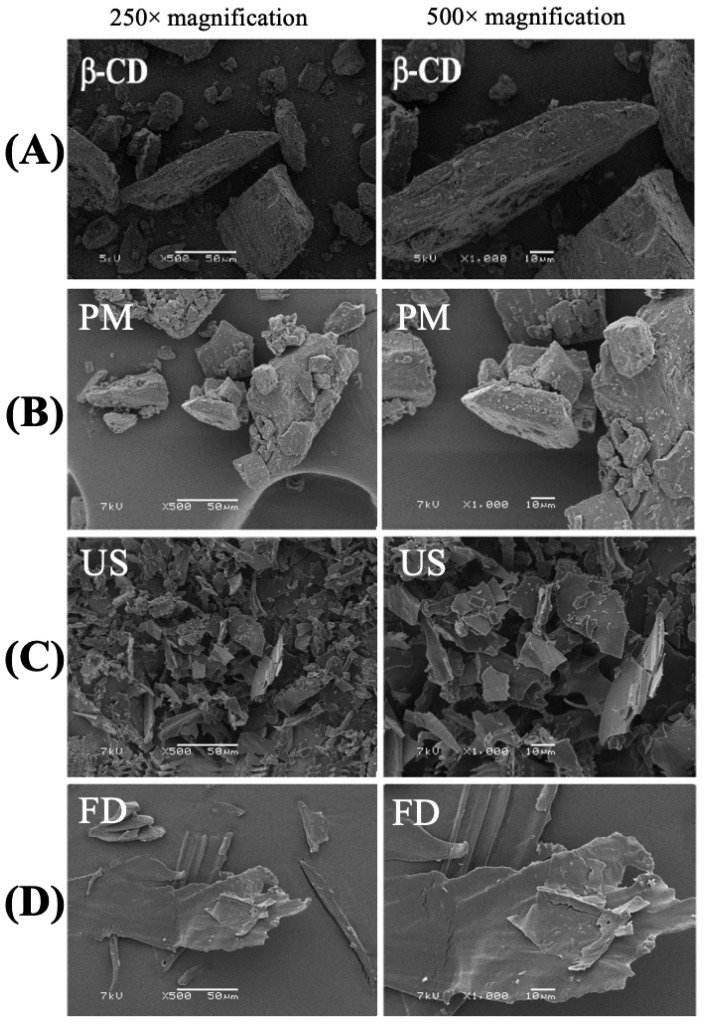
Surface morphology of (**A**) β-CD, (**B**) PM, (**C**) US, and (**D**) FD (magnification 250× and 500×).

The photomicrographs obtained at 250× and 500× magnification (Figure 3) revealed distinct morphological features for β-CD, the PM, and the inclusion complexes. The β-CD displayed well-defined parallelogram-shaped crystals of varying sizes, with smaller particles adhered to their surfaces (Figure 3A), consistent with previous observations [31,32]. The PM and FD samples showed morphological characteristics similar to those of native β-CD, suggesting that the essential oil was primarily adsorbed onto the cyclodextrin surface rather than encapsulated within its cavity (Figure 3B–D). These findings are consistent with the thermal analyses (DSC and TG/DTG), which indicated limited interaction between components [33]. In contrast, the US-prepared complex exhibited a markedly different morphology, characterized by irregularly shaped, amorphous particles with porous surfaces (Figure 3C).

### 3.4. X-Ray Diffraction (XRD)

**Figure 4:**
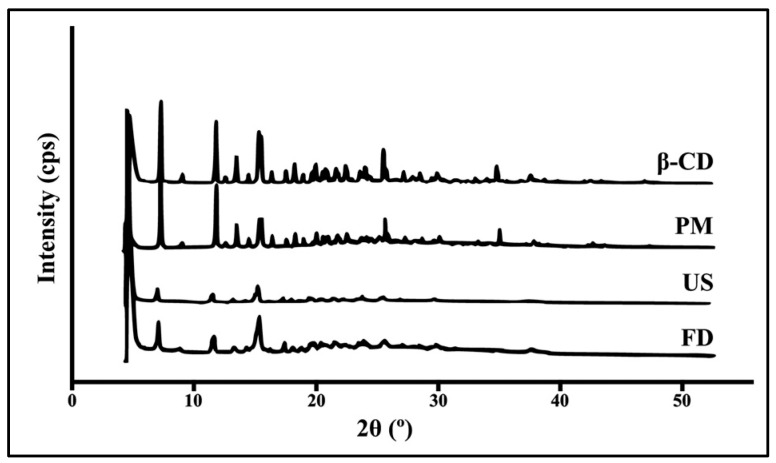
XRD patterns of β-CD, PM, US, and FD.

The X-ray diffractograms show the diffraction patterns of pure β-CD and the inclusion complexes prepared by PM, US and FD (Figure 4). The diffractogram of β-CD showed intense, well-defined peaks, characteristic of its highly crystalline nature and the ordered molecular arrangement within its crystal lattice. The PM sample exhibited diffraction patterns consistent with a simple overlap of β-CD and essential oil peaks, indicating that the β-CD crystalline structure was maintained (Figure 4). In contrast, the US-prepared complex showed a significant decrease in both peak intensity and peak number, reflecting a partial loss of crystallinity. The FD sample exhibited a partial amorphous profile, with the characteristic β-CD peaks either disappearing or showing substantial reduction in intensity (Figure 4) [34,35].

### 3.5. Biocompatibility Assessment of EOCNM and EOCNM/β-CD US Complex

**Figure 5:**
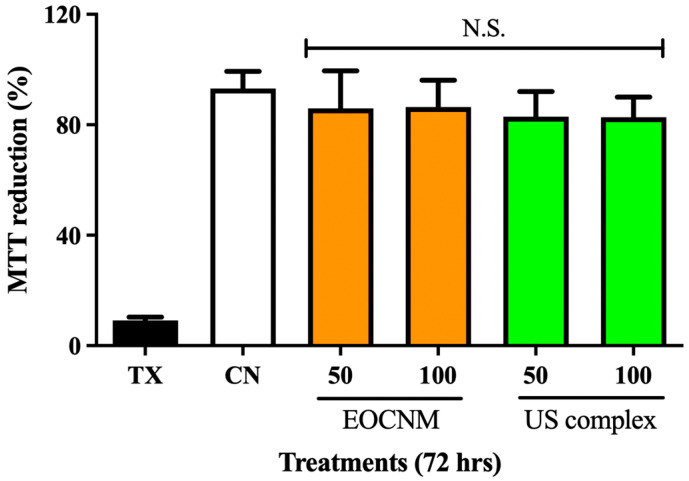
Cell viability of J774 macrophages for TX - Triton X-100 (0.5%) as cytotoxic control; CN as untreated control (cells + DMEM 10% FBS); EOCNM (50 µg·mL^−1^ and 100 µg·mL^−1^); US inclusion complex (50 µg·mL^−1^ and 100 µg·mL^−1^). Data are mean ± SD (*n* = 4). One-way ANOVA/Tukey.

To evaluate the toxicity of the formulated inclusion complexes, J774 macrophage cells were treated with EOCNM and the inclusion complex obtained by the US method at concentrations of 50 and 100 μg·mL^−1^ for 72 h. Cell viability was assessed by MTT reduction, and the results indicate that all treatments maintained viability levels comparable to those of the cells cultured only in DMEM supplemented with 10% fetal bovine serum (FBS), without any treatment and cytotoxic control of cell death (Triton X-100, 0.5% *v*/*v* (Figure 5). Overall, MTT reduction in control cells reached approximately 93%, whereas treated wells ranged between 82 and 85%, with no statistically significant (N.S.) difference between untreated cells and those exposed to EOCNM or the inclusion complex.

## 4. Discussion

In this study, CDs and their inclusion complexes with EOCNM have been widely characterized using thermal analytical techniques due to their fast and informative outputs. Among these techniques, DSC is particularly useful for identifying the formation of inclusion complexes, as evidenced by the reduction, disappearance, or displacement of endothermic or exothermic peaks, as well as by significant changes in the enthalpy of pure or complexed drugs [22].

Our findings demonstrated that β-CD exhibited endothermic events associated with loss of hydration water and thermal degradation, while EOs exhibited events to volatilization and decomposition. This behavior has been related in other studies that evaluated the formation of inclusion complexes between β-CD and EOs [36,37,38]. Furthermore, studies such as those by Andrade et al., who developed inclusion complexes between β-CD and *Hyptis martiusii* Benth essential oil, employed DSC for the characterization of similar systems and observed that PM preserves the thermal peaks of the isolated constituents, indicating an absence of significant complexation [28]. This is because the formation of an inclusion complex is characterized by shifts, attenuation, or disappearance of peaks related to the essential oil, due to its incorporation into the hydrophobic cavity of β-CD and the rearrangement of water originally present in that cavity [22].

The superposition (sum) of the endothermic events of β-CD and EOCNM in the PM thermogram suggests weak intermolecular interactions between the host molecule and the essential oil, indicating low efficiency of this method for inclusion complex formation. Similar findings were reported by Menezes et al. in a study that characterized inclusion complexes between β-CD and *Hyptis pectinata* essential oil [39]. Also, Santos et al. (2025) previously demonstrated a similar mechanism during the complexation of β-CD and the essential oil of farnesol [22].

On the other hand, the complexes prepared by the US and FD methods presented different thermal profiles, due to the absence of typical essential oil peaks in the complex thermogram, which was interpreted as evidence of successful complexation and increased thermal stability of the EO. This observation aligns with previous reports describing the disappearance of melting, volatilization, or sublimation peaks of the guest compound as a strong indicator of inclusion complex formation [15,40]. Thus, the observed results suggest that the EOCNM/β-CD complexation by US method is more thermally stable than the other complexation, according to the DSC technique. Yan et al. (2022) observed similar results when preparing an inclusion complex between β-CD and *Cinnamomum longepaniculatum* essential oil [36]. The absence of endothermic peaks typical of the EO in the thermogram of the complex confirmed that the oil was protected in the hydrophobic cavity of the β-CD.

The DSC technique alone is not enough to ensure the thermal stability of the compounds. Therefore, TG analysis is commonly used in combination with DSC to support the interpretation of calorimetric data, as TG enables the detection of mass changes in the samples as a function of temperature changes [41,42]. In addition, TG allows the assessment of the thermal stability of the formed complexes, as complexation typically alters the decomposition temperatures of the individual components, thereby indicating molecular interactions. Moreover, a slight difference in the temperatures of the mass-loss minima arises from van der Waals forces in the complexes, which are more rapidly disrupted upon heating, while the CD molecule itself decomposes at higher temperatures [43].

TG/DTG analysis revealed distinct thermal behaviors between the EOCNM, β-CD, the PM, and the inclusion complexes. As reported in the study by Santos et al. [22], which obtained inclusion complexes between β-CD and farnesol, essential oils, being mainly composed of low molecular weight compounds, exhibit profiles characterized by mass losses associated primarily with volatilization and subsequent thermal decomposition, a behavior similar to that observed in this study. Similarly, β-CD exhibits a typical multi-step thermal profile, beginning with the loss of hydration water followed by the degradation of the glycopyranoside structure and the formation of carbonaceous residues, a pattern consistent with previously reported data for CDs by Puebla-Duarte et al. and Gupta et al. [44,45]. The overlap of the thermal profile of the PM with that of β-CD confirms that this preparation method does not promote sufficiently strong molecular interactions to significantly alter the thermal behavior of the components, indicating an absence of EO complexation [46,47]. Studies published by Varganici et al., and Hadaruga et al. highlight that in PM, volatile compounds remain adsorbed to the surface of the CD or are free, which is reflected in TG/DTG profiles similar to those of the isolated materials [46,48].

In contrast, the complexes obtained by US and FD showed alterations in the initial mass loss stages, suggesting partial replacement of water molecules present in the β-CD cavity by EO components. This behavior was also observed by other studies, such as those by Lima et al., and Puebla-Duarte et al., which investigated β-CD complexes with EOs, which showed reduced initial mass loss and greater thermal stability when compared to physical mixtures [44,49]. Similar results were obtained by Liu et al. with the production of inclusion complexes between β-CD and *Mosla Chinensis* essential oil using the ultrasound method [37].

Scanning electron microscopy (SEM) analysis allowed us to observe the morphological modifications induced by the different preparation methods and their relationship with the formation of inclusion complexes between β-CD and EOCNM [50,51]. Our results showed that free β-CD presented crystals of varying sizes, rectangular shape, and well-defined surface and contour, as observed by Santos et al. in complexes containing R-(-)-carvone [52]. The micrographs from the PM and FD methods exhibited similar morphology, indicating little interaction between EOCNM and β-CD, as described by Oliveira et al. with inclusion complexes of α-terpineol, a monoterpene alcohol [53]. These findings reinforce that there was no effective complexation, because the morphology of the resulting material reflected that of CD. However, the complex prepared by US presented a distinct morphology, with irregular particles, a partially amorphous aspect, and a porous surface. This loss of crystalline structure suggests strong molecular interactions and the formation of a solid-state inclusion complex between EOCNM and β-CD [54].

Powder X-ray diffraction of crystalline solids is a fundamental technique for elucidating the molecular-level organization of crystalline materials. Consequently, it serves as an essential tool for establishing the relationship between crystal structure and the substance’s physicochemical properties [55]. A detailed understanding of the crystalline structure is therefore critical for interpreting physicochemical characteristics that may influence pharmacological activity [56]. The diffractograms obtained for free β-CD showed intense and well-defined peaks, characteristic of its crystalline nature and the regular organization of its molecules in the crystal lattice [57]. The PM showed overlapping β-CD peaks, without alterations in their intensity or position [36,58]. Veiga et al. [59] obtained similar results when observing interactions of griseofulvin with cyclodextrins using powder X-ray diffractometry, with overlapping peaks, positions, and similar intensity of free β-CD compared to the PM.

However, the complex obtained by the US method showed a reduction in the intensity and number of peaks, indicating a decrease in the degree of crystallinity. This marked decrease in crystallinity supports a more efficient inclusion process, as the essential oil molecules are incorporated within the β-CD cavity, leading to structural reorganization and amorphization of the complex [27,60]. This finding corroborates Abarca et al. [29], who prepared and evaluated complexes between β-CD and 2-nonanone essential oil and observed the disappearance of characteristic peaks and the presence of new diffraction peaks, indicative of the formation of inclusion complexes.

The compound’s molecular properties and the selected preparation method are decisive for the successful formation of cyclodextrin inclusion complexes. The technique employed directly affects the physicochemical performance, morphology, and overall thermal stability of the final formulation. Therefore, comparing different preparation approaches allows the identification of the most suitable method for each specific application [61,62]. Moreover, the preparation process strongly influences the retention of volatile constituents, such as essential oils, which may compromise the physicochemical and thermal stability and effectiveness of the inclusion complex if not adequately controlled [63,64]. Among the available methods, US and FD are advantageous because they minimize or eliminate residual solvents. This is achieved through vacuum-assisted drying or by freezing and sublimation, ensuring cleaner, more stable products [65,66,67]. In contrast, techniques such as paste or slurry complexation may retain traces of solvent, since drying occurs in a desiccator. However, when water is employed as the primary solvent, any residual content is considered non-toxic and does not alter the physicochemical integrity of the resulting complexes [68,69].

The cellular mechanisms underlying the cytotoxicity induced by inclusion complexes have been primarily evaluated using in vitro models, as these systems offer greater control over experimental variables that are more difficult to manipulate in the in vivo assays. Inclusion complexes can be examined in various cell types, with particular emphasis on macrophages, which are the most employed due to their role as the first line of defense of the immune system [70]. Accordingly, in this study, the murine macrophage cell line J774 A.1 was used to assess cytotoxicity in the presence of inclusion complexes and *Cinnamomum camphora* essential oil. Studies report that EOs can induce cytotoxic effects dependent on concentration and exposure time, frequently associated with increased production of reactive oxygen species (ROS), altered mitochondrial activity, DNA damage, and cell death [71,72].

The use of the J774 macrophage lineage is justified because macrophages constitute a central component of the innate immune system and are widely distributed across tissues [73]. The MTT assay is a well-established method to evaluate the biocompatibility and safety of natural and synthetic formulations, as well as for screening potential antitumor or antiviral compounds. This observation is consistent with findings from other studies using extracts of Cinnamomum species, in which MTT assays performed in Vero cells infected with Influenza A H7N3 revealed selective reduction in viability only in infected cells, while maintaining safety in healthy cells [74].

However, the effects vary according to the chemical composition of the oil, cell line, and experimental conditions. In this study, EOCNM and the ultrasound-prepared inclusion complex-maintained cell viability levels similar to the negative control, indicative of low cytotoxicity at the tested concentrations. These findings suggest that, under the tested conditions, the oil components did not trigger relevant cytotoxic responses in J774 A.1 macrophages. Campos et al. [75] reported similar results with free citral essential oil and citral/β-CD inclusion complex in preserving macrophage viability. The absence of cytotoxicity may be related to complexation with β-CD, since the complexation of hydrophobic compounds reduces direct interaction with cell membranes, attenuating irritant or toxic effects [76]. Furthermore, β-CD is widely used due to its biocompatibility in pharmaceutical formulations to promote the safety and stability of active compounds, reinforcing its potential as a safe delivery matrix [22,77]. Additionally, studies report that some camphor-rich oils, such as that of *C. camphora*, exhibit low toxicity to normal cells at moderate concentrations. On the other hand, more pronounced cytotoxic effects occurred in infected or tumor cells [72,78,79]. Thus, the results indicate the safety and low cytotoxicity of EOCNM and the inclusion complex in murine macrophages.

## 5. Conclusions

Thermal (DSC, TG/DTG), morphological (SEM), and structural (XRD) analyses collectively confirm the successful formation of an inclusion complex between EOCNM and β-CD via US-assisted complexation. The disappearance or displacement of characteristic thermal events associated with free EOCNM, together with the modified crystalline profile observed in the XRD diffractogram of the complex, provides strong evidence of molecular encapsulation within the β-CD cavity. Likewise, SEM micrographs reveal a distinct morphological reorganization in the complexes compared with the physical mixture and individual components, further indicating effective interaction.

The encapsulated formulation demonstrated enhanced physicochemical and thermal stability, including a higher onset of thermal degradation and reduced mass loss rate, supporting the protective role of β-CD against volatilization and thermal decomposition of the essential oil. These properties highlight the suitability of cyclodextrin-based systems for improving the handling, stability, and potential bioavailability of volatile and labile natural products. Biological evaluation using the MTT reduction assay showed that the EOCNM:β-CD inclusion complex did not significantly alter the cytotoxic profile of EOCNM in J774 macrophages, with cell viability comparable to that observed for the free EO and control cells. These results indicate that microencapsulation does not exacerbate cytotoxic effects, but also does not confer additional improvements in biocompatibility under the conditions evaluated.

Thus, the combined physicochemical and in vitro data support β-CD complexation as an efficient and scalable strategy to enhance the physicochemical and thermal stability, safety, and technological applicability of EOCNM. These findings provide a solid foundation for further development of β-CD-based delivery systems for essential oils in pharmaceutical, cosmetic, and food formulations, as well as for future studies aimed at optimizing release kinetics, biological activity, and stability under different environmental conditions.

## Figures and Tables

**Table 1:** Mass loss percentage obtained by TG/DTG of EOCNM, β-CD, PM, US and FD (Mean ± SD).

Sample	Δm_1_ (%)	Δm_2_ (%)	Δm_3_ (%)	Δm_4_ (%)	Degradation
	30–100 °C	100–360 °C	360–600 °C	600–900 °C	Temperature °C
β-CD	13.1 ± 0.22	74.6 ± 0.35	10.0 ± 0.19	1.5 ± 0.20	290 ± 2.5
EOCNM	3.5 ± 0.19	75.5 ± 0.32	14.4 ± 0.11	1.0 ± 0.12	260 ± 3.2
PM	13.4 ± 0.25	72.0 ± 0.27	4.6 ± 0.14	0.2 ± 0.13	268 ± 2.8
US	11.1 ± 0.18	80.0 ± 0.43	5.0 ± 0.21	2.6 ± 0.22	280 ± 1.9
FD	11.9 ± 0.24	74.0 ± 0.36	2.4 ± 0.18	0.5 ± 0.18	260 ± 4.0

## Data Availability

Data is included within this article.

## Abbreviations

The following abbreviations are used in this manuscript:

β-CD	β-cyclodextrin
CD	Cyclodextrin
CNM	*Cinnamomum camphora*
DTG	Derivative thermogravimetry
DSC	Differential scanning calorimetry
DMEM	Dulbecco’s Modified Eagle Medium
EO	Essential oil
EOCNM	*Cinnamomum camphora* essential oil
FD	Freeze-drying
NS	Not significant
PM	Physical mixing
SEM	Scanning electron microscopy
TG	Thermogravimetric
TX	Triton X-100
US	Ultrasonic
XRD	X-ray diffraction

## References

[1] Prabhuji S.K., Rao G.P., Pande S., Richa, Srivastava G.K., Srivastava A.K. (2021). Cinnamomum Species: Spices of Immense Medicinal and Pharmacological Values. Med. Plants Int. J. Phytomed. Relat. Ind..

[2] Tien Cuong N., Ngoc Dai D., Van Chung M., Hong Ban P. (2021). The Diversity of Lauraceae Family in Pu Huong Nature Reserve, Nghe an Province. Vinh Univ. J. Sci..

[3] Huang J.F., Li L., van der Werff H., Li H.W., Rohwer J.G., Crayn D.M., Meng H.H., van der Merwe M., Conran J.G., Li J. (2016). Origins and Evolution of Cinnamon and Camphor: A Phylogenetic and Historical Biogeographical Analysis of the *Cinnamomum* Group (Lauraceae). Mol. Phylogenet. Evol..

[4] Zhang C., Fan L., Fan S., Wang J., Luo T., Tang Y., Chen Z., Yu L. (2019). *Cinnamomum cassia* Presl: A Review of Its Traditional Uses, Phytochemistry, Pharmacology and Toxicology. Molecules.

[5] Wei X., Li G.-H., Wang X.-L., He J.-X., Wang X.-N., Ren D.-M., Lou H.-X., Shen T. (2017). Chemical Constituents from the Leaves of *Cinnamomum parthenoxylon* (Jack) Meisn. (Lauraceae). Biochem. Syst. Ecol..

[6] Guoruoluo Y., Zhou H., Wang W., Zhou J., Aisa H.A., Yao G. (2018). Chemical Constituents from the Immature Buds of *Cinnamomum cassia* (Lauraceae). Biochem. Syst. Ecol..

[7] Zhou H., Ren J., Li Z. (2017). Antibacterial Activity and Mechanism of Pinoresinol from *Cinnamomum camphora* Leaves against Food-Related Bacteria. Food Control.

[8] Stancu A.I., Mititelu M., Ficai A., Ditu L.-M., Buleandră M., Badea I.A., Pincu E., Stoian M.C., Brîncoveanu O., Boldeiu A. (2025). Comparative Evaluation of β-Cyclodextrin Inclusion Complexes with Eugenol, Eucalyptol, and Clove Essential Oil: Characterisation and Antimicrobial Activity Assessment for Pharmaceutical Applications. Pharmaceutics.

[9] Bianchini N.H., Gouveia F.N., Da Silveira M.F., Pinheiro C.G., Heinzmann B.M. (2022). Essential Oils of Blepharocalyx, Nectandra, and Piper: Activity Against Wood-Rotting Fungi. Nativa.

[10] Carvalho Silva L., Franco Carvalhedo L., Costa Vieira J.P., de Carvalho Silva L.A., dos Santos Monteiro O., Araújodo Carmo L.H. (2015). Delineamento de Formulações Cosméticas Com Óleo Essencial de Lippia Gracilis Schum (Alecrim-DeTabuleiro) de Origem Amazônica. J. Basic Appl. Pharm. Sci..

[11] Zhang D., Lv P., Zhou C., Zhao Y., Liao X., Yang B. (2019). Cyclodextrin-Based Delivery Systems for Cancer Treatment. Mater. Sci. Eng. C.

[12] Santos E.H., Kamimura J.A., Hill L.E., Gomes C.L. (2015). Characterization of Carvacrol Beta-Cyclodextrin Inclusion Complexes as Delivery Systems for Antibacterial and Antioxidant Applications. LWT Food Sci. Technol..

[13] Paiva-Santos A.C., Ferreira L., Peixoto D., Silva F., Soares M.J., Zeinali M., Zafar H., Mascarenhas-Melo F., Raza F., Mazzola P.G. (2022). Cyclodextrins as an Encapsulation Molecular Strategy for Volatile Organic Compounds—Pharmaceutical Applications. Colloids Surf. B Biointerfaces.

[14] Benkő B.-M., Tóth G., Moldvai D., Kádár S., Szabó E., Szabó Z.-I., Kraszni M., Szente L., Fiser B., Sebestyén A. (2024). Cyclodextrin Encapsulation Enabling the Anticancer Repositioning of Disulfiram: Preparation, Analytical and in Vitro Biological Characterization of the Inclusion Complexes. Int. J. Pharm..

[15] Serafini M.R., Menezes P.P., Costa L.P., Lima C.M., Quintans L.J., Cardoso J.C., Matos J.R., Soares-Sobrinho J.L., Grangeiro S., Nunes P.S. (2012). Interaction of P-Cymene with β-Cyclodextrin. J. Therm. Anal. Calorim..

[16] Costa S., Barreto I.C., Gama N., Santos K., Oliveira C.M., de Costa I.S., Vila Nova M., Santos R., Borges A., de Alencar Filho J.M.T. (2025). Patent-Based Technological Overview of Propolis–Cyclodextrin Inclusion Complexes with Pharmaceutical Potential. Pharmaceutics.

[17] Bertici R.A., Ridichie A., Bertici N.S., Ledeţi A., Ledeţi I., Văruţ R.-M., Sbârcea L., Albu P., Rădulescu M., Rusu G. (2025). Compatibility Studies of Sildenafil-HPBCD Inclusion Complex with Pharmaceutical Excipients. Pharmaceutics.

[18] Pârvănescu R.D., Păpurică M., Alexa I.O., Dehelean C.A., Șoica C., Moacă E.A., Ledeți A., Voicu M., Coricovac D., Trandafirescu C. (2025). The Potential of Amphiphilic Cyclodextrins as Carriers for Therapeutic Purposes: A Short Overview. Pharmaceutics.

[19] Nicolaescu O.E., Ionescu C., Samide A., Tigae C., Spînu C.I., Oprea B. (2025). Advancements in Cyclodextrin Complexes with Bioactive Secondary Metabolites and Their Pharmaceutical Applications. Pharmaceutics.

[20] Nicolaescu O.E., Belu I., Mocanu A.G., Manda V.C., Rău G., Pîrvu A.S., Ionescu C., Ciulu-Costinescu F., Popescu M., Ciocîlteu M.V. (2025). Cyclodextrins: Enhancing Drug Delivery, Solubility and Bioavailability for Modern Therapeutics. Pharmaceutics.

[21] Su J., Chen J., Li L., Li B., Shi L., Zhang H., Ding X. (2012). Preparation of Natural Borneol/2-Hydroxypropyl-β-Cyclodextrin Inclusion Complex and Its Effect on the Absorption of Tetramethylpyrazine Phosphate in Mouse. Chem. Pharm. Bull..

[22] Santos A.M., Vieira E.M., de Jesus J.R., Santana Júnior C.C., Nascimento Júnior J.A.C., Oliveira A.M.S., Araújo A.A.d.S., Picot L., Alves I.A., Serafini M.R. (2025). Development and Characterization of Farnesol Complexed in β- and Hydroxypropyl-β-Cyclodextrin and Their Antibacterial Activity. Carbohydr. Res..

[23] Wang H., Wang S., Zhu H., Wang S., Xing J. (2019). Inclusion Complexes of Lycopene and β-Cyclodextrin: Preparation, Characterization, Stability and Antioxidant Activity. Antioxidants.

[24] Kim J.-S. (2020). Study of Flavonoid/Hydroxypropyl-β-Cyclodextrin Inclusion Complexes by UV-Vis, FT-IR, DSC, and X-Ray Diffraction Analysis. Prev. Nutr. Food Sci..

[25] Carmichael J., DeGraff W.G., Gazdar A.F., Minna J.D., Mitchell J.B. (1987). Evaluation of a Tetrazolium-Based Semiautomated Colorimetric Assay: Assessment of Chemosensitivity Testing. Cancer Res..

[26] Grela E., Ząbek A., Grabowiecka A. (2015). Interferences in the Optimization of the MTT Assay for Viability Estimation of Proteus Mirabilis. Avicenna J. Med. Biotechnol..

[27] Herrera A., Rodríguez F.J., Bruna J.E., Abarca R.L., Galotto M.J., Guarda A., Mascayano C., Sandoval-Yáñez C., Padula M., Felipe F.R.S. (2019). Antifungal and Physicochemical Properties of Inclusion Complexes Based on β-Cyclodextrin and Essential Oil Derivatives. Food Res. Int..

[28] Andrade T.A., Freitas T.S., Araújo F.O., Menezes P.P., Dória G.A.A., Rabelo A.S., Quintans-Júnior L.J., Santos M.R.V., Bezerra D.P., Serafini M.R. (2017). Physico-Chemical Characterization and Antibacterial Activity of Inclusion Complexes of *Hyptis martiusii* Benth Essential Oil in β-Cyclodextrin. Biomed. Pharmacother..

[29] Abarca R.L., Rodríguez F.J., Guarda A., Galotto M.J., Bruna J.E. (2016). Characterization of Beta-Cyclodextrin Inclusion Complexes Containing an Essential Oil Component. Food Chem..

[30] Trindade G.G.G., Thrivikraman G., Menezes P.P., França C.M., Lima B.S., Carvalho Y.M.B.G., Souza E.P.B.S.S., Duarte M.C., Shanmugam S., Quintans-Júnior L.J. (2019). Carvacrol/β-Cyclodextrin Inclusion Complex Inhibits Cell Proliferation and Migration of Prostate Cancer Cells. Food Chem. Toxicol..

[31] Abou-Okeil A., Rehan M., El-Sawy S.M., El-bisi M.K., Ahmed-Farid O.A., Abdel-Mohdy F.A. (2018). Lidocaine/β-Cyclodextrin Inclusion Complex as Drug Delivery System. Eur. Polym. J..

[32] Hădărugă N.G. (2012). *Ficaria verna*Huds. Extracts and Their β-Cyclodextrin Supramolecular Systems. Chem. Cent. J..

[33] Menezes P.P., Serafini M.R., Quintans-Júnior L.J., Silva G.F., Oliveira J.F., Carvalho F.M.S., Souza J.C.C., Matos J.R., Alves P.B., Matos I.L. (2014). Inclusion Complex of (−)-Linalool and β-Cyclodextrin. J. Therm. Anal. Calorim..

[34] Luo X., Zeng L., Li Q., Wang Z., Kong F., Bi Y. (2022). β-Cyclodextrin Inclusion Complex Containing Essential Oil from Wampee [*Clausena lansium* (Lour.) Skeels] Fruit Pericarp: Synthesis, Characterization, and Evaluation of Antioxidant Activity. J. Mol. Struct..

[35] Cui H., Siva S., Lin L. (2019). Ultrasound Processed Cuminaldehyde/2-Hydroxypropyl-β-Cyclodextrin Inclusion Complex: Preparation, Characterization and Antibacterial Activity. Ultrason. Sonochem..

[36] Yan Y., Zhao X., Wang C., Fang Q., Zhong L., Wei Q. (2022). Preparation, Optimization, and Characterization of Inclusion Complexes of *Cinnamomum longepaniculatum* Essential Oil in β-Cyclodextrin. Sustainability.

[37] Liu H.-N., Jiang X.-X., Naeem A., Chen F.-C., Wang L., Liu Y.-X., Li Z., Ming L.-S. (2022). Fabrication and Characterization of β-Cyclodextrin/*Mosla chinensis* Essential Oil Inclusion Complexes: Experimental Design and Molecular Modeling. Molecules.

[38] Wu H., Jiang X., Dong Z., Fan Q., Huang J., Liu H., Chen L., Li Z., Ming L. (2024). New Insights into the Influence of Encapsulation Materials on the Feasibility of Ultrasonic-Assisted Encapsulation of *Mosla chinensis* Essential Oil. Ultrason. Sonochem..

[39] Menezes P., Araujo A., Doria G., Quintans-Junior L., Oliveira M., dos Santos M., Oliveira J., Matos J., Carvalho F., Alves P. (2015). Physicochemical Characterization and Analgesic Effect of Inclusion Complexes of Essential Oil from *Hyptis pectinata* L. Poit Leaves with β-Cyclodextrin. Curr. Pharm. Biotechnol..

[40] Ruz Sanjuan V., Van den Mooter G., Carlos I.Z., dos Santos Ramos M.A., Bauab T.M., Tercini A.C.B., González Bedia M.M., Gomes de Oliveira A. (2020). Stability, Biological and Biopharmaceutical Evaluation of the Inclusion Complexes of the Antifungal and Antiprotozoal Drug Candidate 2-(2-Nitrovinyl) Furan (G-0) with Beta Cyclodextrin Derivatives. J. Drug Deliv. Sci. Technol..

[41] Zhu X., Ping W. (2014). Optimization of β-Cyclodextrin Cross-Linked Polymer for Monitoring of Quercetin. Spectrochim. Acta Part A Mol. Biomol. Spectrosc..

[42] Zheng F., Li M., Li C., Zhou B., Xuan X., Li H. (2023). Wireless Surface Acoustic Wave Humidity Sensor with Chitosan/Porous Cyclodextrin–TiO_2_ Composites for Monitoring Air and Human Respiration. Sens. Actuators B Chem..

[43] Burkeev M., Fazylov S., Bakirova R., Iskineyeva A., Sarsenbekova A., Tazhbaev E., Davrenbekov S. (2021). Thermal Decomposition of β-Cyclodextrin and Its Inclusion Complex with Vitamin E. Mendeleev Commun..

[44] Puebla-Duarte A.L., Bernal-Mercado A.T., Santos-Sauceda I., Acosta-Elias M., Fernández-Quiroz D., Burruel-Ibarra S.E., Ornelas-Paz J.D.J., Pérez-Cabral I.D., Rodríguez-Félix F., Iturralde-García R.D. (2025). The Characterization and Antioxidant and Erythroprotective Effects of β-Carotene Complexed in β-Cyclodextrin. Int. J. Mol. Sci..

[45] Gupta G.R., Patil P.D., Shaikh V.R., Kolhapurkar R.R., Dagade D.H., Patil K.J. (2018). Analytical Estimation of Water Contents, Specific Heat Capacity and Thermal Profiles Associated with Enzymatic Model Compound β-Cyclodextrin. Curr. Sci..

[46] Hădărugă N.G., Bandur G.N., David I., Hădărugă D.I. (2019). A Review on Thermal Analyses of Cyclodextrins and Cyclodextrin Complexes. Environ. Chem. Lett..

[47] Durante M., Milano F., De Caroli M., Giotta L., Piro G., Mita G., Frigione M., Lenucci M.S. (2020). Tomato Oil Encapsulation by α-, β-, and γ-Cyclodextrins: A Comparative Study on the Formation of Supramolecular Structures, Antioxidant Activity, and Carotenoid Stability. Foods.

[48] Varganici C.-D., Marangoci N., Rosu L., Barbu-Mic C., Rosu D., Pinteala M., Simionescu B.C. (2015). TGA/DTA–FTIR–MS Coupling as Analytical Tool for Confirming Inclusion Complexes Occurrence in Supramolecular Host–Guest Architectures. J. Anal. Appl. Pyrolysis.

[49] dos Santos Lima B., de Alcântara Campos C., da Silva Santos A.C.R., Santos V.C.N., Trindade G.D.G.G., Shanmugam S., Pereira E.W.M., Marreto R.N., Duarte M.C., da Silva Almeida J.R.G. (2019). Development of Morin/Hydroxypropyl-β-Cyclodextrin Inclusion Complex: Enhancement of Bioavailability, Antihyperalgesic and Anti-Inflammatory Effects. Food Chem. Toxicol..

[50] Dan Córdoba A.V., Aiassa V., Longhi M.R., Quevedo M.A., Zoppi A. (2020). Improved Activity of Rifampicin Against Biofilms of *Staphylococcus aureus* by Multicomponent Complexation. AAPS PharmSciTech.

[51] Mendes C., Wiemes B.P., Buttchevitz A., Christ A.P., Ribas K.G., Adams A.I.H., Silva M.A.S., Oliveira P.R. (2015). Investigation of β-Cyclodextrin–Norfloxacin Inclusion Complexes. Part 1. Preparation, Physicochemical and Microbiological Characterization. Expert Rev. Anti Infect. Ther..

[52] Santos A.M., Nascimento Júnior J.A.C., Oliveira A.M.S., Santana Júnior C.C., de Carvalho Neto A.G., Falcão M.A.P., Walker C.I.B., de Souza Carvalho F.M., de Aquino T.M., Quintans-Júnior L.J. (2025). R-(-)-Carvone/β-Cyclodextrin Inclusion Complexes: Physicochemical Profiling and Embryotoxicity Evaluation in a Zebrafish Model. Supramol. Chem..

[53] Oliveira M.G.B., Brito R.G., Santos P.L., Araújo-Filho H.G., Quintans J.S.S., Menezes P.P., Serafini M.R., Carvalho Y.M.B.G., Silva J.C., Almeida J.R.G.S. (2016). α-Terpineol, a Monoterpene Alcohol, Complexed with β-Cyclodextrin Exerts Antihyperalgesic Effect in Animal Model for Fibromyalgia Aided with Docking Study. Chem. Biol. Interact..

[54] Anaya-Castro M.A., Ayala-Zavala J.F., Muñoz-Castellanos L., Hernández-Ochoa L., Peydecastaing J., Durrieu V. (2017). β-Cyclodextrin Inclusion Complexes Containing Clove (*Eugenia caryophyllata*) and Mexican Oregano (*Lippia berlandieri*) Essential Oils: Preparation, Physicochemical and Antimicrobial Characterization. Food Packag. Shelf Life.

[55] Thakral N.K., Zanon R.L., Kelly R.C., Thakral S. (2018). Applications of Powder X-Ray Diffraction in Small Molecule Pharmaceuticals: Achievements and Aspirations. J. Pharm. Sci..

[56] Braga D., Casali L., Grepioni F. (2022). The Relevance of Crystal Forms in the Pharmaceutical Field: Sword of Damocles or Innovation Tools?. Int. J. Mol. Sci..

[57] Hasegawa K., Goto S., Tsunoda C., Kuroda C., Okumura Y., Hiroshige R., Wada-Hirai A., Shimizu S., Yokoyama H., Tsuchida T. (2023). Using Singular Value Decomposition to Analyze Drug/β-Cyclodextrin Mixtures: Insights from X-Ray Powder Diffraction Patterns. Phys. Chem. Chem. Phys..

[58] Wang Y., Yin C., Cheng X., Li G., Shan Y., Zhu X. (2020). β-Cyclodextrin Inclusion Complex Containing *Litsea cubeba* Essential Oil: Preparation, Optimization, Physicochemical, and Antifungal Characterization. Coatings.

[59] Veiga M.D., Díaz P.J., Ahsan F. (1998). Interactions of Griseofulvin with Cyclodextrins in Solid Binary Systems. J. Pharm. Sci..

[60] Marques C.S., Carvalho S.G., Bertoli L.D., Villanova J.C.O., Pinheiro P.F., dos Santos D.C.M., Yoshida M.I., de Freitas J.C.C., Cipriano D.F., Bernardes P.C. (2019). β-Cyclodextrin Inclusion Complexes with Essential Oils: Obtention, Characterization, Antimicrobial Activity and Potential Application for Food Preservative Sachets. Food Res. Int..

[61] Cid-Samamed A., Rakmai J., Mejuto J.C., Simal-Gandara J., Astray G. (2022). Cyclodextrins Inclusion Complex: Preparation Methods, Analytical Techniques and Food Industry Applications. Food Chem..

[62] Ghatbandhe N.M., Sangole P.A., Punyaprediwar N.D., Kavale A.K., Satpute G.D. (2024). Methods of Preparation and Characterization of Cyclodextrin Encapsulated Inclusion Complex: Review. Int. J. Sci. Res. Sci. Technol..

[63] Zhang X., Su J., Wang X., Wang X., Liu R., Fu X., Li Y., Xue J., Li X., Zhang R. (2022). Preparation and Properties of Cyclodextrin Inclusion Complexes of Hyperoside. Molecules.

[64] Rudrangi S.R.S., Bhomia R., Trivedi V., Vine G.J., Mitchell J.C., Alexander B.D., Wicks S.R. (2015). Influence of the Preparation Method on the Physicochemical Properties of Indomethacin and Methyl-β-Cyclodextrin Complexes. Int. J. Pharm..

[65] Lakeh M.A., Karimvand S.K., Khoshayand M.R., Abdollahi H. (2020). Analysis of Residual Moisture in a Freeze-Dried Sample Drug Using a Multivariate Fitting Regression Model. Microchem. J..

[66] Jakubowska E., Bielejewski M., Milanowski B., Lulek J. (2022). Freeze-Drying of Drug Nanosuspension–Study of Formulation and Processing Factors for the Optimization and Characterization of Redispersible Cilostazol Nanocrystals. J. Drug Deliv. Sci. Technol..

[67] Resende de Azevedo J., Espitalier F., Ré M.I. (2019). Ultrasound Assisted Crystallization of a New Cardioactive Prototype Using Ionic Liquid as Solvent. Ultrason. Sonochem..

[68] Umamaheswari D., Gupta N.T., Kumar M., Venkateswarlu B.S. (2021). A Review on—Estimation of Residual Solvents by Different Analytical Method. Int. J. Pharm. Sci. Rev. Res..

[69] Banchero M. (2021). Supercritical Carbon Dioxide as a Green Alternative to Achieve Drug Complexation with Cyclodextrins. Pharmaceuticals.

[70] De Godoy K.F., Rodolpho J.M.D.A., Fragelli B.D.D.L., Camillo L., Brassolatti P., Assis M., Nogueira C.T., Speglich C., Longo E., Anibal F.D.F. (2022). Cytotoxic Effects Caused by Functionalized Carbon Nanotube in Murine Macrophages. Cell. Physiol. Biochem..

[71] Bakkali F., Averbeck S., Averbeck D., Idaomar M. (2008). Biological Effects of Essential Oils—A Review. Food Chem. Toxicol..

[72] Cherneva E., Pavlovic V., Smelcerovic A., Yancheva D. (2012). The Effect of Camphor and Borneol on Rat Thymocyte Viability and Oxidative Stress. Molecules.

[73] Sharma D.C., Shukla R., Ali J., Sharma S., Bajpai P., Pathak N. (2016). Phytochemical Evaluation, Antioxidant Assay, Antibacterial Activity and Determination of Cell Viability (J774 and THP1 Alpha Cell Lines) of *P. sylvestris* Leaf Crude and Methanol Purified Fractions. EXCLI J..

[74] Fatima M., Zaidi N.-S.S., Amraiz D., Afzal F. (2016). In Vitro Antiviral Activity of *Cinnamomum cassia* and Its Nanoparticles Against H7N3 Influenza A Virus. J. Microbiol. Biotechnol..

[75] Campos C.A., Lima B.S., Trindade G.G.G., Souza E.P.B.S.S., Mota D.S.A., Heimfarth L., Quintans J.S.S., Quintans-Júnior L.J., Sussuchi E.M., Sarmento V.H.V. (2019). Anti-Hyperalgesic and Anti-Inflammatory Effects of Citral with β-Cyclodextrin and Hydroxypropyl-β-Cyclodextrin Inclusion Complexes in Animal Models. Life Sci..

[76] Kumar S., Pooja, Trotta F., Rao R. (2018). Encapsulation of Babchi Oil in Cyclodextrin-Based Nanosponges: Physicochemical Characterization, Photodegradation, and In Vitro Cytotoxicity Studies. Pharmaceutics.

[77] Júnior J.A.C.N., Santos A.M., Oliveira A.M.S., Santos A.B., de Souza Araújo A.A., Frank L.A., Serafini M.R. (2024). Use of Nanotechnology Applied to Sunscreens: Technological Prospection Based on Patents. J. Drug Deliv. Sci. Technol..

[78] Celen Yuceturk S., Aydogan Turkoglu S., Kockar F., Kucukbay F.Z., Azaz A.D. (2021). Essential Oil Chemical Composition, Antimicrobial, Anticancer, and Antioxidant Effects of *Thymus convolutus* Klokov in Turkey. Z. Naturforschung C.

[79] Yener I., Yilmaz M.A., Olmez O.T., Akdeniz M., Tekin F., Hasimi N., Alkan M.H., Ozturk M., Ertas A. (2020). A Detailed Biological and Chemical Investigation of Sixteen *Achillea* Species’ Essential Oils via Chemometric Approach. Chem. Biodivers..

